# Functional Coupling of Duplex Translocation to DNA Cleavage in a Type I Restriction Enzyme

**DOI:** 10.1371/journal.pone.0128700

**Published:** 2015-06-03

**Authors:** Eva Csefalvay, Mikalai Lapkouski, Alena Guzanova, Ladislav Csefalvay, Tatsiana Baikova, Igor Shevelev, Vitali Bialevich, Katsiaryna Shamayeva, Pavel Janscak, Ivana Kuta Smatanova, Santosh Panjikar, Jannette Carey, Marie Weiserova, Rüdiger Ettrich

**Affiliations:** 1 Center for Nanobiology and Structural Biology, Institute of Microbiology and Global Change Research Center, Academy of Sciences of the Czech Republic, Zamek 136, CZ-373 33 Nove Hrady, Czech Republic; 2 Institute of Microbiology, Academy of Sciences of the Czech Republic, Vídeňská 1083, 142 20 Praha 4, Czech Republic; 3 Faculty of Sciences, University of South Bohemia in Ceske Budejovice, Zamek 136, CZ-373 33 Nove Hrady, Czech Republic; 4 Institute of Molecular Genetics, Academy of Sciences of the Czech Republic, Vídeňská 1083, 142 20 Praha 4, Czech Republic; 5 Institute of Molecular Cancer Research, University of Zürich, Wintherthurerstrasse 190, CH-8057 Zürich, Switzerland; 6 Australian Synchrotron, 800 Blackburn Road, Clayton VIC 3168, Australia; 7 Department of Biochemistry and Molecular Biology, Monash University, Clayton Campus, Melbourne, VIC 3800 Australia; 8 Chemistry Department, Princeton University, Princeton, New Jersey 08544–1009, United States of America; Institute of Enzymology of the Hungarian Academy of Science, HUNGARY

## Abstract

Type I restriction-modification enzymes are multifunctional heteromeric complexes with DNA cleavage and ATP-dependent DNA translocation activities located on motor subunit HsdR. Functional coupling of DNA cleavage and translocation is a hallmark of the Type I restriction systems that is consistent with their proposed role in horizontal gene transfer. DNA cleavage occurs at nonspecific sites distant from the cognate recognition sequence, apparently triggered by stalled translocation. The X-ray crystal structure of the complete HsdR subunit from *E*. *coli* plasmid R124 suggested that the triggering mechanism involves interdomain contacts mediated by ATP. In the present work, *in vivo* and *in vitro* activity assays and crystal structures of three mutants of EcoR124I HsdR designed to probe this mechanism are reported. The results indicate that interdomain engagement *via* ATP is indeed responsible for signal transmission between the endonuclease and helicase domains of the motor subunit. A previously identified sequence motif that is shared by the RecB nucleases and some Type I endonucleases is implicated in signaling.

## Introduction

Restriction-modification (RM) systems protect bacteria against foreign DNA [1–3]. Unmodified invading DNA can be eliminated by restriction activity, which introduces a double-strand break, while modification-methylase activity protects the cell’s own DNA from cleavage. Type I RM enzymes house these activities in a multisubunit complex having ATP-dependent translocation activity that pulls thousands of duplex DNA base pairs through the stationary enzyme, eventually cleaving at random sites distant from the recognition site [4–6]. Type I enzymes are found in over half of all bacterial genomes, including many pathogens [7,8]. The seemingly redundant but ultimately random cleavage by Type I enzymes has been suggested to have a role in horizontal gene transfer [9]. Their peculiar activities have attracted much biochemical attention that has begun to shed light on their mechanisms.

Typical Type I complexes comprise five subunits encoded by the *hsd* (*h*ost *s*pecificity of *D*NA) genes [10–13]. One specificity subunit HsdS recognizes a specific, short, usually asymmetric DNA sequence, and anchors the enzyme complex there. Two HsdM subunits housing the methylation activity that marks host DNA bind to the DNA-HsdS complex. Upon identification of an unmethylated specific DNA sequence, two HsdR motor subunits, which bear the endonuclease and ATP-dependent DNA translocation activities in the fully assembled DNA-HsdS-HsdM_2_-HsdR_2_ complex, start to translocate the duplex DNA toward the stationary enzyme from both directions, producing two extruded DNA loops flanking the enzyme complex [14,15]. One enzyme complex anchored at a single site can translocate an entire circular DNA prior to cleavage [11], whereas linear DNAs presenting more than one enzyme-binding site can be cleaved [6,13].

It has long been thought that endonuclease activation requires an event that blocks duplex translocation [6, 16–19]. A Holliday junction [20] or a replication fork that is then cleaved at the branch [21] can block translocation. Collision of two enzyme complexes [6] or, on circular DNAs, translocation of the entire circle [17,19] can also act as a block. Blocked translocation appears to trigger transition of the enzyme complex to an endonucleolytic mode, suggesting that translocation and DNA cleavage are alternative processes in Type I enzymes. Transition from a translocating mode to an endonucleolytic mode implies signal transmission within the motor subunit, but its mechanism has been unclear.

**Fig 1:**
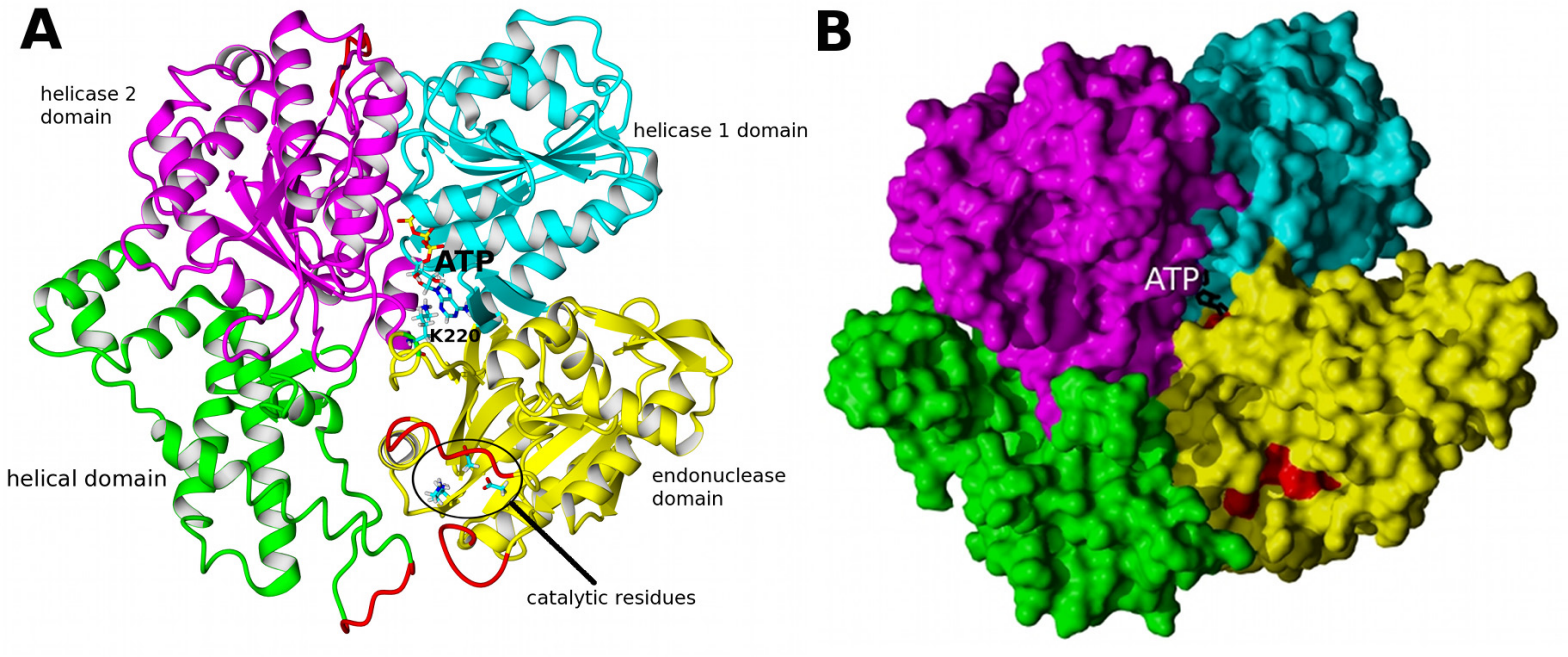
EcoR124I motor subunit HsdR. A, Structure of the motor subunit as reported in [22]. The four domains are color-coded: yellow endonuclease, cyan helicase 1, magenta helicase 2 (which together form the translocase); green helical. The structure comprises residues 13–892 of the polypeptide sequence of native HsdR, and is identical with the WT crystal structure 2W00 except for the parts shown in red which were modeled (142–147, 182–189, 585–590, and 859–869) as they are unresolved in the WT crystal structure. ATP is shown as a skeletal model with cyan carbons. Selected side chains relevant for this work are also indicated as skeletal models: Lys220, which contacts ATP, as well as the three conserved catalytic site residues (Asp151, Lys165, and Glu167) are shown in multiple colors with cyan carbons. B, Four domains form a planar array shown as a space-fill cartoon. The four domains are color-coded as in A. Duplex DNA is thought to follow a path down the center of this ‘face’ of the motor subunit, contacting all four domains. Red shading of the endonuclease active site and residue Lys220 that contacts ATP (black skeletal model) emphasizes that the two regions are ~20 Å apart.

The X-ray crystal structure of the HsdR subunit with bound ATP from the Type I restriction enzyme of *E*. *coli* plasmid R124 [22] revealed four structural and functional domains forming a planar array (Fig 1): one nuclease domain of PD-(E/D)xK type [23,24] that houses three endonuclease active-site residues [25] and a QxxxY motif common to the RecB-like nucleases [26–28]; two RecA-like helicase domains of SF2 type [29,30] that use ATP to translocate duplex DNA without unwinding [31]; and a C-terminal α-helix-rich domain presumed to contact the HsdS-HsdM_2_ methylase. ATP bound between the two helicase domains was found unexpectedly to also contact Lys220 of the endonuclease domain, suggesting a means by which nuclease and translocation activities might communicate.

The present work aimed to evaluate the role of residue 220 in EcoR124I function by mutagenesis, crystallization, and activity tests *in vivo* and *in vitro*. Early in the course of this work one mutant revealed the first resolved QxxxY motif, lying directly above the endonuclease active site. That finding enabled molecular dynamics simulations (MD) of the behavior of both the QxxxY motif and the region of residue 220 [32]. The MD results suggested that the contact of Lys220 with ATP alternates with a contact to the QxxxY motif, and that large-scale domain motions are correlated with these alternative contacts. In that context the results of the present work indicate why positive charge at residue 220 is required for full function.

## Results

### *In vivo* activity

The restriction phenotype of three mutants of HsdR (Lys220Ala, Lys220Glu, Lys220Arg) was determined *in vivo* by testing the ability of cells producing each mutant, or the WT HsdR, to restrict the growth of unmodified bacteriophage lambda in the presence of the WT DNA methyltransferase. Previously described positive and negative complementation tests [33] were used to distinguish between defects in DNA cleavage and defects in interaction with the HsdS-HsdM_2_ methyltransferase, respectively. For these complementation tests (described in Methods) restriction-proficient JM109(DE3)/pKF650 r^+^m^+^ or restriction-deficient JM109(DE3)/pACMS r^-^m^+^ cells were transformed with the plasmid pTrcR124 [11] expressing the WT or mutated *hsdR* gene, and the efficiency of plating of lambda phage on these strains was determined (Table 1).

**Table 1:** Effect of changes at HsdR Lys220 on the restriction phenotype of EcoR124I.

	Restriction activity ^a^	
HsdR	r− host ^b^	r+ host^c^
WT	0.0025 ± 0.0026^SD^	0.00019 ± 0.000056
Lys220Arg	0.0014 ± 0.0014	0.00029 ± 0.000071
Lys220Ala	0.031 ± 0.0015	0.0012 ± 0.00028
Lys220Glu	0.27 ± 0.041	0.045 ± 0.034

^a^ Restriction activity was determined as the efficiency of plating of λvir.0 on the test strains relative to the efficiency of plating of λvir.0 on *E*. *coli* JM109(DE3) indicator (nonrestricting) strain as described in Methods. The values are the mean of at least three independent experiments. ^SD^ The standard deviation

^b^ Positive complementation was tested in r− host *E*. *coli* JM109(DE3)[pACMS] (r−m+).

^c^ Negative complementation was tested in r+ host *E*. *coli* JM109(DE3)[pKF650] (r+m+).

The results of the positive complementation test in the r^-^ host reveal that relative to WT HsdR, restriction is reduced ~10-fold by mutant Lys220Ala and ~100-fold by Lys220Glu. Both the Lys220Arg HsdR mutant subunit and the WT HsdR subunit confer a restriction-proficient phenotype in the r^-^ host, suggesting that efficient DNA cleavage requires a positive charge at position 220. In the negative complementation test in the r+ host, a mutant HsdR subunit that is altered in its ability to cleave DNA but is competent for assembly with methyltransferase can compete in subunit assembly with WT HsdR subunits produced in the r+ host, reducing restriction activity. This test, also called the trans-dominance test, establishes that a mutant subunit is capable of subunit assembly. A mutant subunit that reduces the restriction activity of the WT subunit produced in the r^+^ host also corroborates a restriction-deficient mutant phenotype found in the r^-^ host. Lys220Ala or Lys220Glu mutant HsdR subunits display a trans-dominant effect that is reflected in a 5- or 400-fold reduction, respectively, of restriction in the r^+^ host (Table 1), consistent with their reduced restriction in the r^-^ host. Both WT and Lys220Arg HsdR show no trans-dominant effect in the r^+^ host, consistent with their restriction proficiency in the r^-^ host. Taken together, the *in vivo* results indicate that none of the mutant motor subunits is defective in interacting with the methyltransferase to form the endonuclease complex, and that Lys220 located far from the endonuclease active site exerts a profound effect on the restriction activity of the enzyme.

### *In vitro* DNA cleavage and binding

**Fig 2:**
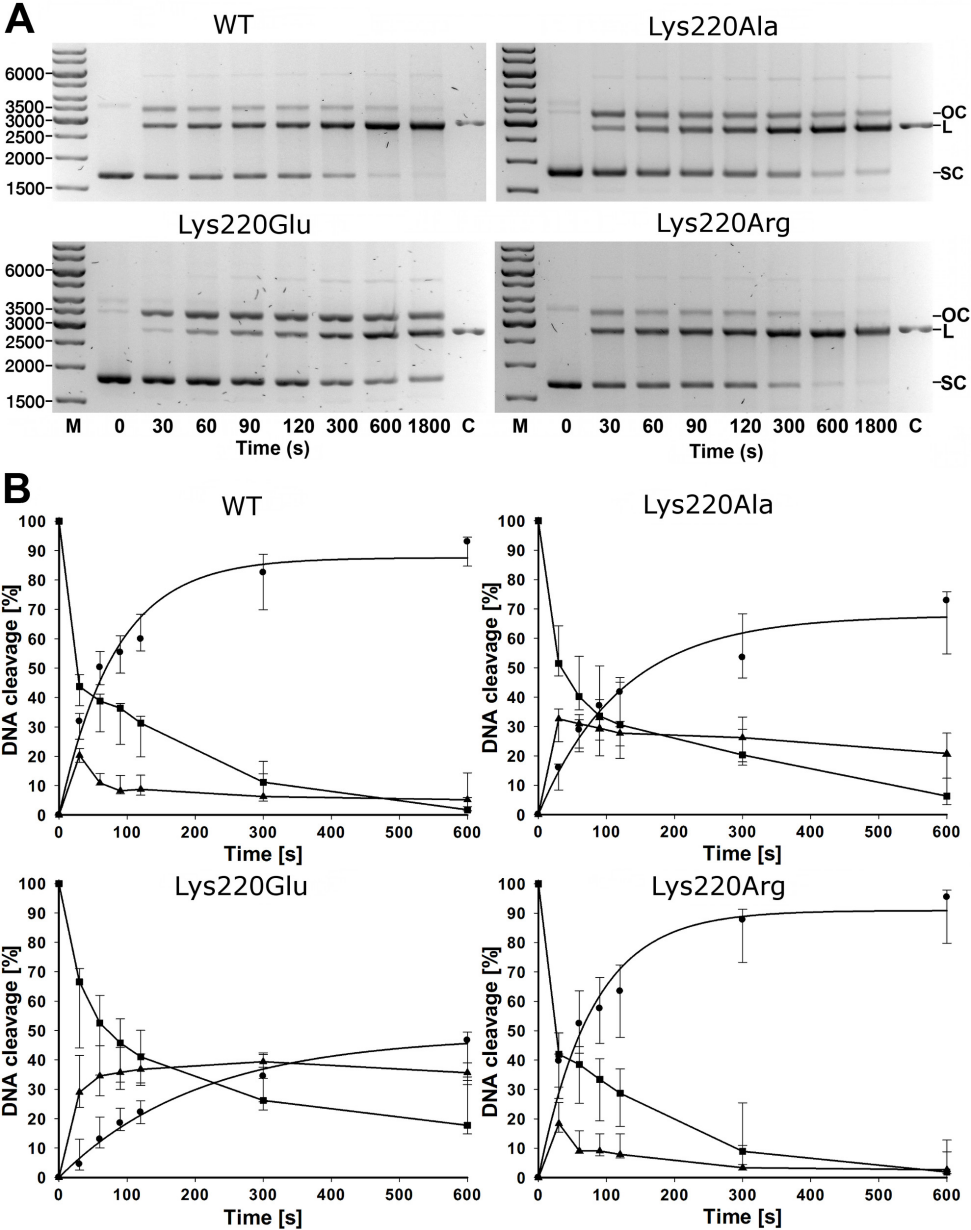
Cleavage of circular DNA. Circular plasmid DNA bearing one EcoR124I recognition site was reacted with enzymes reconstituted from HsdS_1_HsdM_2_ methylase and WT HsdR or mutant HsdRs Lys220Glu, Lys220Ala, or Lys220Arg, and analyzed as in Fig 2. OC, open circular product (▲); L, linear product (●); SC, supercoiled substrate (■); C, control plasmid linearized with HindIII. A: Reactions stopped at the indicated time points were applied to 1.2% agarose gels and visualized by ethidium bromide staining. M is the marker of the indicated numbers of basepairs and C is the linearized plasmind DNA as a control B: Quantification. The three indicated DNA species were quantified individually. Plots for the increase of linear DNA product were derived by fitting an exponential rise to maximum function in SigmaPlot. The points are given for quantification of the gels shown in A, and standard deviations are given from the mean of seven repetitions (WT, Lys220Ala, Lys220Glu) or six repetitions (Lys220Arg) of the experiment conducted with independently purified enzyme preparations.

DNA cleavage activity of the HsdR mutants *in vitro* was evaluated using covalently closed circular plasmid DNA containing a single EcoR124I recognition site (Fig 2). The time point in Fig 2B at which circular DNA substrate concentration equals the linearized DNA product concentration is referred to as crossover time and is taken as an inverse measure of enzyme activity. Crossover time for WT EcoR124I is ~50 seconds using a ratio of HsdR to methylase (6:1) chosen to yield maximum enzyme activity; additional HsdR does not increase activity. Under these conditions the Lys220Arg mutant displays nearly WT activity with a crossover time of ~40–50 sec. Mutant Lys220Ala is approximately half as active as WT and mutant Lys220Glu approximately one-third as active, with crossover times of ~90–100 and ~150 sec, respectively.

Rate constants for the appearance of linearized DNA were estimated by fitting an exponential rise to maximum to the data of Fig 2. The best-fit values of the rate constant λ are 0.0122 s−1 and 0.0127 s−1 for WT and mutant Lys220Arg, respectively, whereas λ values for Lys220Ala and Lys220Glu are substantially lower at 0.0081 s−1 and 0.0048 s−1, respectively. Thus the crossover times and rate constants indicate that the rank order of enzyme activity in vitro is Lys220Arg>WT> Lys220Ala>Lys220Glu. These activities follow the same quantitative order as the *in vivo* phage restriction results.

**Fig 3:**
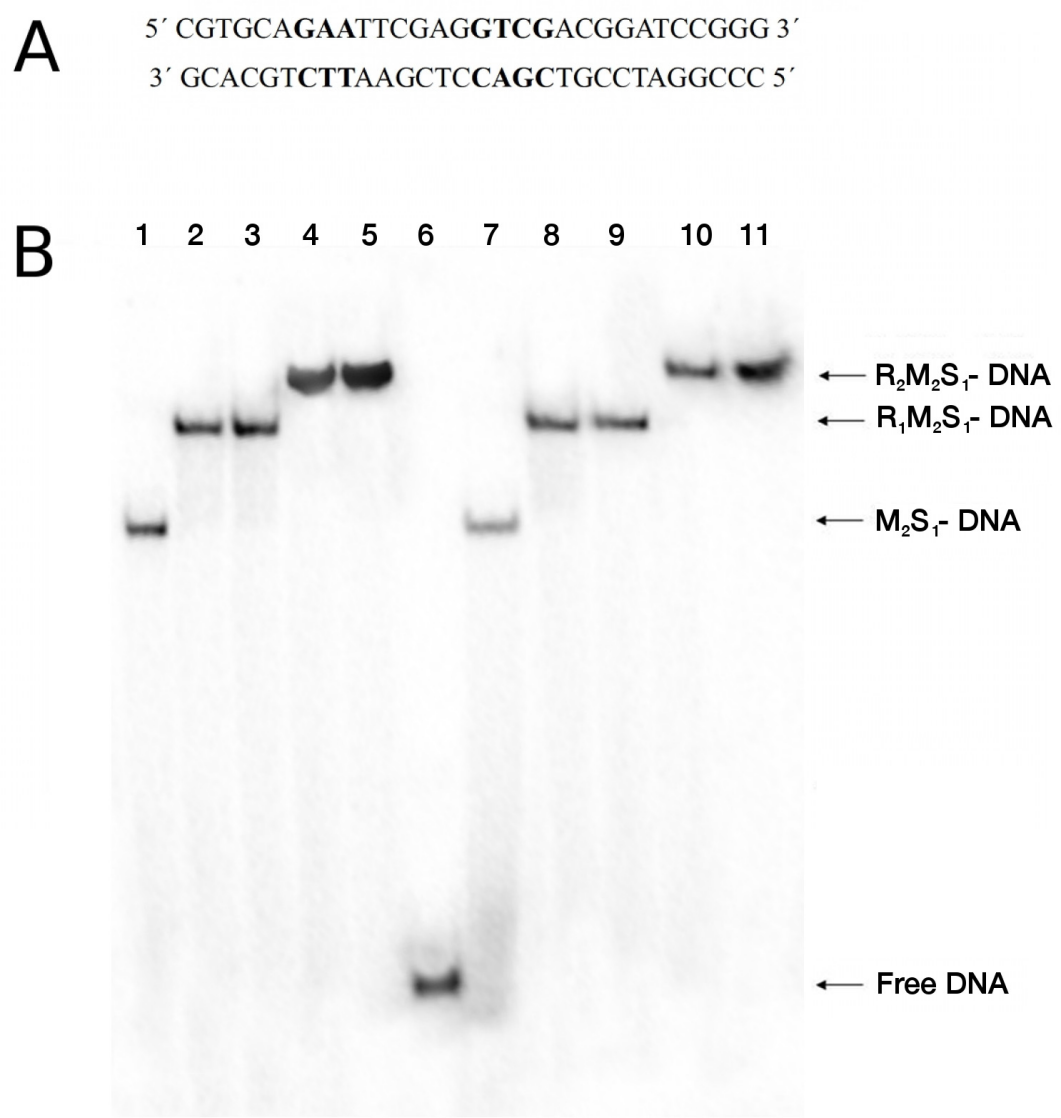
DNA binding. A. Sequence of synthetic 30-basepair oligonucleotide used, with the EcoR124I recognition sequence shown in bold. B. Electrophoretic mobility-shift assay. The oligonucleotide was 5’ end-labeled with polynucleotide kinase and titrated with EcoR124I reconstituted from HsdS_1_HsdM_2_ methylase and WT or Lys220Glu HsdR. The oligonucleotide concentration is 5 nM, the concentration of methylase (M2S1 complex) is 40nM, and the concentrations of HsdR are 20, 40, 80, and 120 nM, respectively, in lanes 2–5 (WT) and 8–11 (Lys220Glu). Lane 6, DNA only; lanes 1 and 7, DNA and methylase only. The numbers of subunits in each DNA-protein complex are indicated on the right: R, motor subunit HsdR; M, methylase subunit HsdM; S, specificity subunit HsdS.

To determine if changes in DNA binding contribute to the altered restriction activities of the mutants, DNA binding by the most defective mutant enzyme, Lys220Glu, was compared with WT in an electrophoretic mobility-shift assay [34, 26] using an oligonucleotide bearing the recognition sequence (Fig 3). Fig 3 shows that no significant differences are evident in forming the complexes with one or two motor subunits in the absence of ATP, indicating that DNA binding is unaltered even in the mutant that is most defective in the *in vivo* and *in vitro* restriction assays. Thus, the results indicate that the reduced cleavage activity of the Lys220Glu HsdR mutant and the Lys220Ala mutant (data not shown), is not due to defective DNA binding.

### ATPase activity

**Fig 4:**
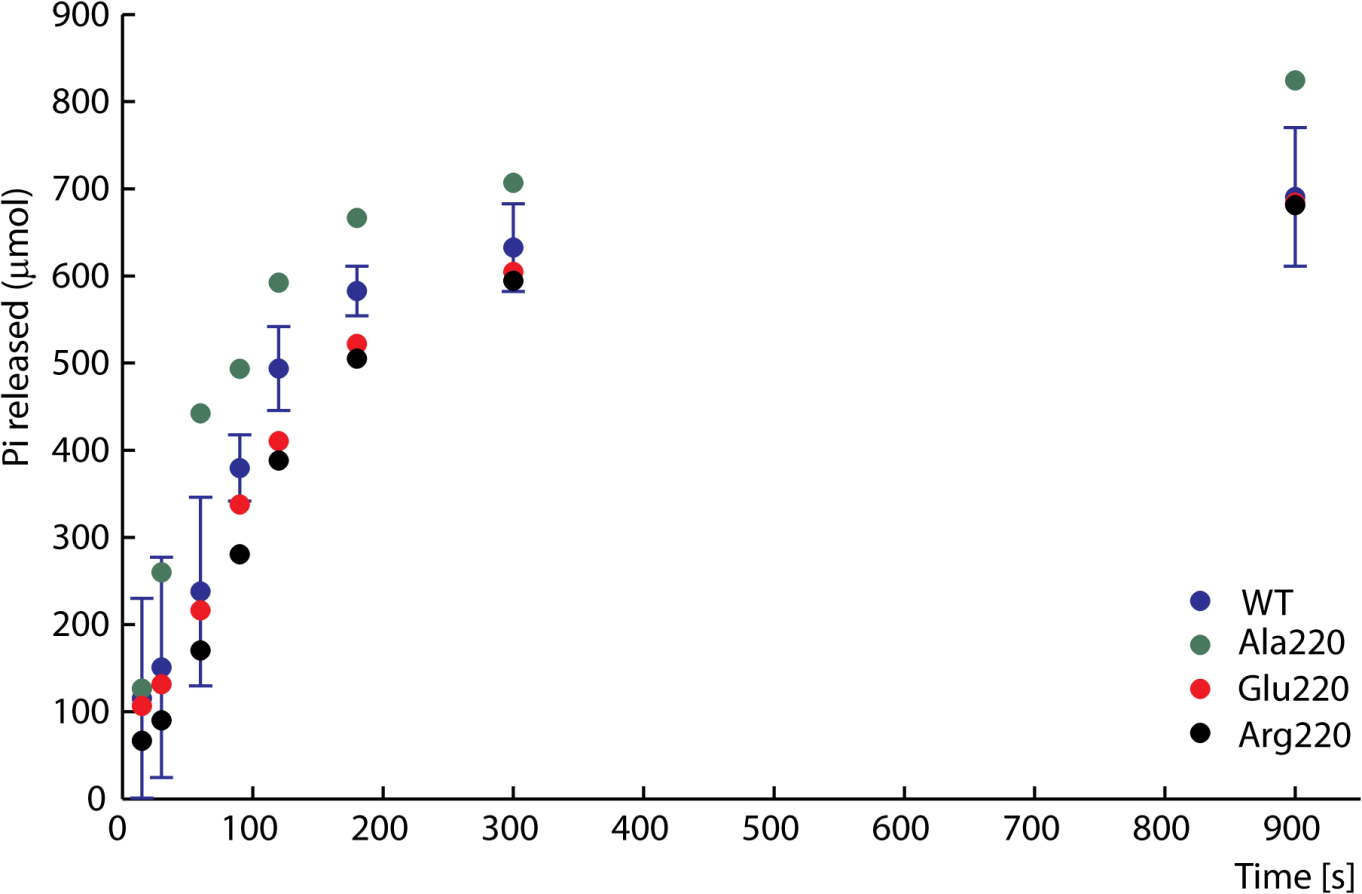
DNA-dependent ATPase activity. EcoR124I reconstituted from methylase and WT (blue) or mutant HsdRs Lys220Ala (green), Lys220Glu (red), or Lys220Arg (black) was incubated at a final concentration of 15 nM with 90 nM circular plasmid DNA containing one recognition site and 2 mM ATP containing 0.16 μCi g-^32^P-ATP. At the indicated time points ATP and inorganic phosphate were resolved on cellulose TLC, autoradiographed, and scanned to quantify the extent of hydrolysis. For clarity error bars are shown for WT only as they would overlay, nevertheless they are of similar dimension for all mutants.

WT EcoR124I hydrolyses approximately one ATP per base pair translocated [35]. The DNA-dependent ATPase hydrolysis rate is considered to be related to the rate of translocation [33]. ATPase activities of the purified WT and mutant HsdR subunits were measured after reconstitution with HsdS-HsdM_2_ methylase and addition of covalently closed circular plasmid DNA containing a single EcoR124I recognition site. Commonly used ATPase assays with malachite green [11] or TLC detection of radioactive phosphate [36, 37] gave similar results (data not shown). In control experiments without DNA the ATP hydrolysis rate in the radioactivity assay was found to be no higher than that of ATP alone in solution (data not shown). Results obtained at saturating ATP concentrations (2 mM ATP, Fig 4) are typical of those obtained at ATP concentrations in the range 0.1 to ~3 mM. The results for the WT enzyme reproduce its previously reported ATPase activity [11]. The ATPase activities of the three mutant enzymes are all very similar to that of WT EcoR124I, with initial rates estimated by a linear fit to the first few time points of 3.155 ± 0.534 μM/s for WT, 3.551 ± 0.035 μM/s for Ala 220, 3.253 ± 0.476 μM/s for Glu220 and 3.2105 ± 0.238 μM/s for Arg220. This result indicates that Lys220 plays little or no direct role in ATP hydrolysis despite contacting the edge of the adenine base. Even the Lys220Glu mutant with greatly reduced endonuclease activity maintains WT-like ATPase activity.

### DNA translocation

**Fig 5:**
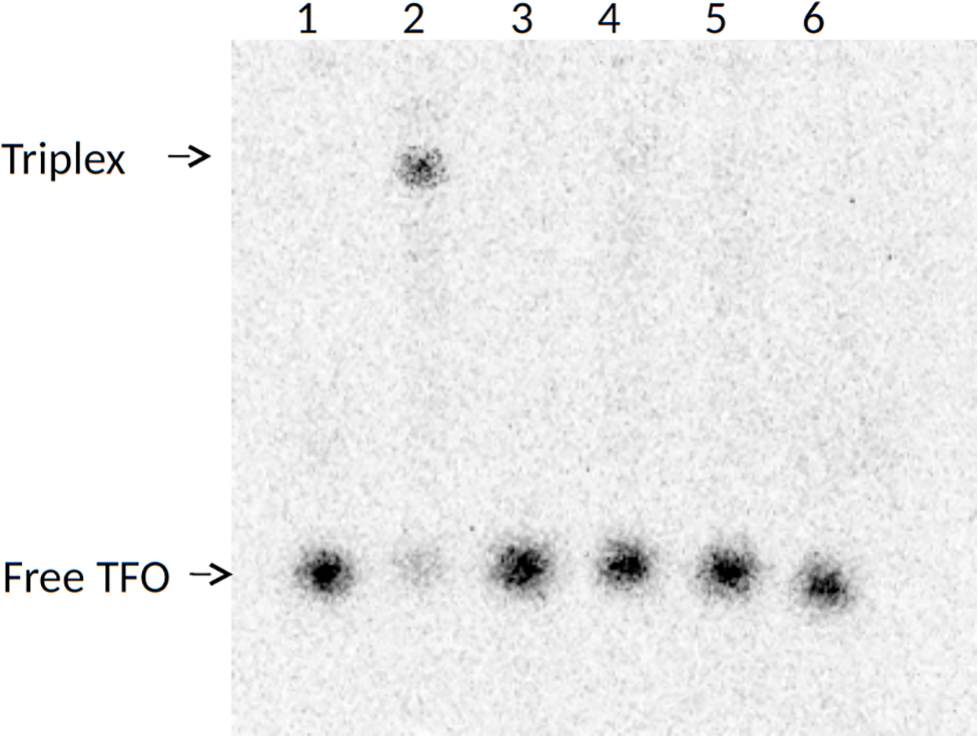
DNA translocation analysis by triplex displacement. Reactions were examined by agarose gel electrophoresis and autoradiography of ^32^P-labelled-triplex-forming-oligonucleotide (Materials and Methods). Bound (triplex) and free triplex-forming-oligonucleotide (TFO) are indicated. Lane 1, free TFO; lane 2, triplex formed by the triplex-forming-oligonucleotide and covalently closed pLKS5 plasmid DNA; lane 3, triplex incubated with endonuclease reconstituted with wildtype HsdR; lane 4, triplex incubated with endonuclease reconstituted with mutant Lys220Ala HsdR, lane 5, triplex incubated with endonuclease reconstituted with mutant Lys220Glu HsdR; triplex incubated with endonuclease reconstituted with mutant Lys220Arg HsdR.

Translocation of duplex DNA by reconstituted Lys220Ala, Lys220Glu, and Lys220Arg mutant and WT enzymes was measured using a triplex displacement assay [38,39]. In this assay, an oligonucleotide forms a DNA triplex at a specific location and is displaced when the DNA passes through the enzyme during translocation. Each enzyme complex was reconstituted using methylase and the respective mutant or WT HsdR in the same ratio (1:2.5). Enzyme to DNA ratios (10:1) and reaction times (1h) were also identical for the mutant and WT enzymes. The agarose gel in Fig 5 shows that the triplex-forming oligonucleotide is fully displaced from the triplex by each mutant or WT enzyme complex, indicating that all mutant enzymes are capable of DNA translocation. Thus the observed reduction of restriction function of even the most defective mutant, Lys220Glu, is not due to an inability to translocate DNA.

These functional results can be summarized as follows. All mutant enzymes bind methylase *in vivo* and *in vitro*; all mutant enzymes bind DNA, hydrolyze ATP, and translocate duplex DNA; and all mutant enzymes cut circular DNA bearing one recognition site similarly as WT. WT and Lys220Arg are r^+^ *in vivo*, Lys220Ala is ~10% r^+^ and Lys220Glu is r^-^, in agreement with the relative order of *in vitro* restriction activity, which is WT≈Lys220Arg>Lys220Ala>Lys220Glu. Thus it is only nuclease activity that is specifically impaired by mutations at position 220, despite the location of this site ~20Å away from the endonuclease active site.

### Structures of mutant HsdR subunits

The three mutant HsdR subunits were crystallized with bound ATP, and their X-ray crystal structures were refined at 2.74 Å, 2.84 Å, and 2.99 Å (Table 2) for the Lys220Arg, Lys220Ala, and Lys220Glu mutants, respectively, guided by the higher-resolution structure of the WT HsdR-ATP complex (2.6 Å; 22). The crystallization conditions used previously for WT HsdR yielded crystals only for the Lys220Arg mutant; the Lys220Ala and Lys220Glu mutants required altered conditions (Methods). The overall structure of all four HsdRs in these crystals is the same, although the structures of the Lys220Arg and Lys220Ala mutants present only two molecules in the asymmetric unit whereas Lys220Glu has four; WT HsdR crystals have two molecules in the asymmetric unit. The four crystal structures differ in the region around residue 220 as discussed below. As the overall structure of each mutant HsdR is essentially identical to WT, the observed differences described here are likely to result from the mutations and not from the differences in lattice contacts.

**Table 2:** Crystallographic data collection and refinement statistics.

	Lys220Arg HsdR	Lys220Glu HsdR	Lys220Ala HsdR
**Data collection**			
Space group	P2_1_	P2_1_	P2_1_
Cell dimensions			
*a*, *b*, *c* (Å)	87.05, 124.35, 128.01	127.11, 123.11, 160.11	86.33, 124.49, 128.76
*β* (°)	108.86	111.48	108.31
Resolution (Å)	68.0–2.72 (2.87–2.72)*	20.0–2.99 (3.17–2.99)	18.9–2.84 (2.99–2.84)
*R*_merge_ (%)	15.9 (33.2)	11.6 (40.1)	9.1 (59.4)
*I* / s*I*	7.1 (1.9)	10.7(3.7)	13.5 (2.3)
Completeness (%)	91.5 (67.9)	98.8 (96.2)	97.6 (87.6)
Redundancy	2.3 (2.1)	4.2 (4.1)	3.8 (3.5)
**Refinement**			
Resolution (Å)	32.36–2.74	19.89–2.99	18.86–2.84
No. reflections	62959	91171	56464
*R*_work_/ *R*_free_	25.17 / 29.23	25.55 / 29.65	23.36 / 28.04
No. atoms	13388	27369	13761
Protein	13226	27241	13689
ATP/Mg/PO_4_	62/2/20	124/4/0	62/2/0
Water	78	0	8
*B*-factors			
Protein	39.75	54.50	54.00
ATP/Mg/PO_4_	20.19/24.11/42.21	41.71/50.7/-	31.55/48.66/-
Water	23.90	0	49.55
R.m.s. deviations			
Bond lengths (Å)	0.004	0.004	0.010
Bond angles (°)	1.06	1.00	1.27
PDB ID	4BE7	4BEB	4BEC

* Values in parentheses are for the highest-resolution shell.

**Fig 6:**
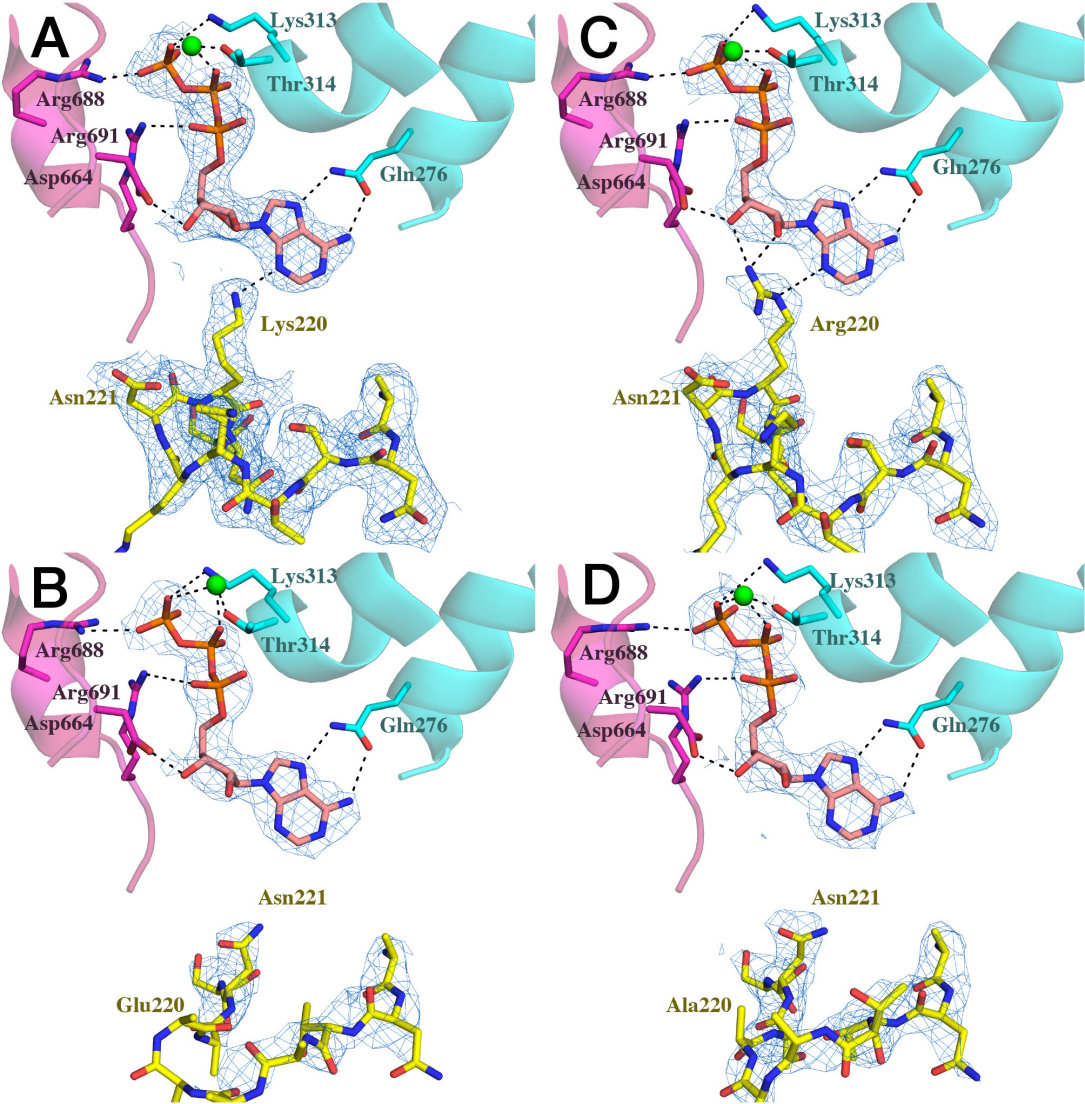
ATP contacts. Models and electron density are shown for A, WT HsdR; B, Lys220Glu chain A; C, Lys220Arg; D, Lys220Ala. Domain segments (ribbons) and selected residues (stick models) are color-coded as in Fig 1, with Mg ion shown as a green sphere. Electron density (blue mesh) is shown for ATP (upper center of each panel, atomic colors and orange carbon) and for the 220s loop (lower). The electron density for WT HsdR is better at the same contour level due to its higher resolution, with corresponding differences in the electron density mesh spacing. Dashed lines indicate distances short enough to permit bonding interactions between the indicated functional groups.

The position and orientation of ATP is the same in all four HsdR structures, with identical contacts observed between ATP and Arg688, Arg691, Asp664, Gln276, Thr314, and Lys313. Fig 6 compares the ATP-binding sites of WT and the three mutant HsdRs in the same orientation as in Fig 1A. In the WT endonuclease domain Lys220 lies near the N-terminus of α8, with its ε-NH_2_ nitrogen atom 3.1 Å from N3 on the edge of the adenine base. In the Lys220Arg mutant the ε-NH nitrogen atom lies 3.7 Å from adenine N3, bringing its proton almost to hydrogen-bonding distance with the base edge. The closer of the two equivalent ω-NH_2_ nitrogens lies 3.1 Å from ribose 3'-OH and 2.6 Å from ribose 2'-OH, enabling one or more potential hydrogen bonds with the ribose. In contrast, the Lys220 NH_2_ nitrogen atom of WT HsdR lies 3.6 Å from ribose 2'-OH and 4.6 Å from ribose 3'-OH. Thus, Arg substitutes structurally for the Lys220 interaction with the ATP base edge, and can make a novel interaction with the ribose as well.

Residue 220 is located at the base of a loop made by residues 215–219 that is well-resolved only in the WT and Lys220Arg mutant structures (Fig 6A and 6C). The main chain can be traced for residues 217–219 in the Lys220Ala mutant, though with less confidence than in the WT or Lys220Arg structures (Fig 6D). In the Lys220Glu mutant (Fig 6B) the loop is less defined than in the Lys220Ala structure, although main-chain atoms of residues 216–220 could be modeled. Thus the ordering of this loop may be facilitated by hydrogen-bonding of Lys220 or Arg220 to ATP, which is lacking in the Lys220Ala and Lys220Glu mutants. Adjacent to the loop Asn221 is well-resolved in all four structures, but its location and sidechain conformation differ. Residues 220 and 221 are on one turn of a short helix-like segment. In the WT and Lys220Arg structures, where residue 220 contacts ATP, Asn221 points away from ATP. In the Lys220Ala and Lys220Glu structures, where the residue 220 sidechain does not form a hydrogen bond with ATP, the helix-like segment is rotated so as to bring the alpha carbon of Asn221 into the position of the alpha carbon of residue 220 in the WT and Lys220Arg structures. In these two structures the Asn221 sidechain points toward ATP and partially fills the space that in the other two structures is occupied by the sidechain of residue 220, but the Asn221 sidechain does not approach the ATP base edge closely enough to hydrogen bond as Lys220 or Arg220 can. The entire region encompassing the 215–219 loop and the following short helix-like segment is hereafter referred to as the 220s loop.

**Fig 7:**
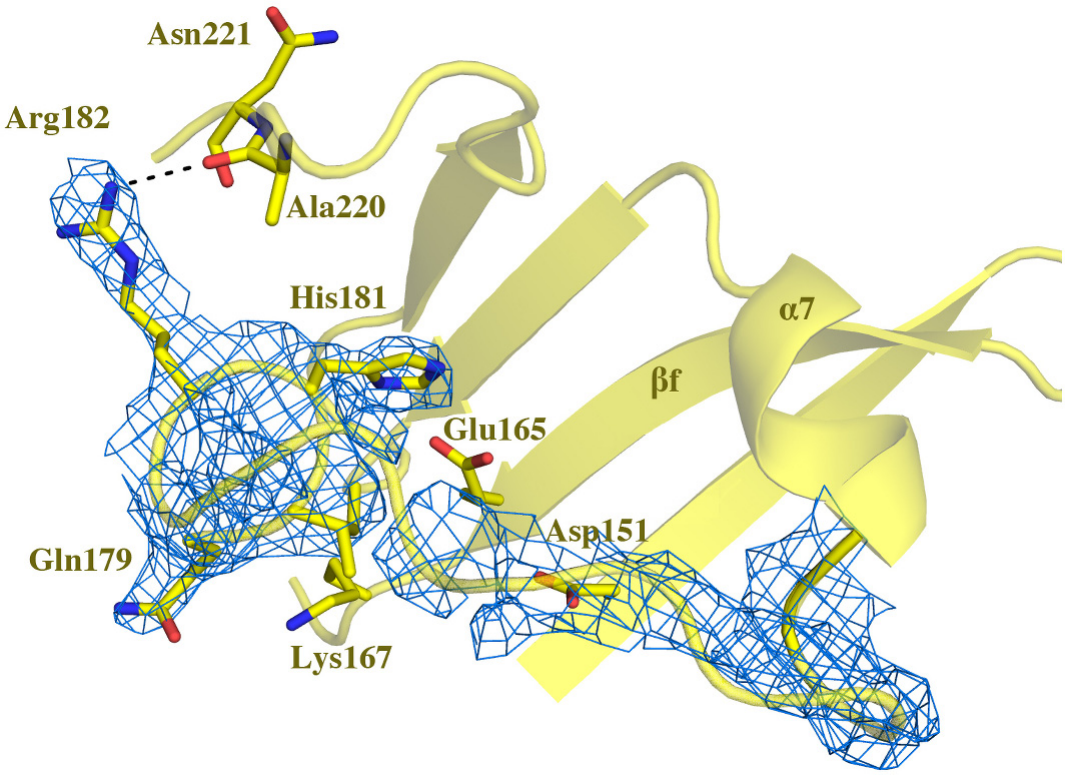
Resolved 180s loop. Electron density (blue mesh) in Lys220Ala mutant HsdR is shown only for the 180s loop, with selected sidechains of the loop shown as sticks in atomic colors with yellow carbons. Outside the 180s loop the sidechains of residues Asp151, Glu165, and Lys167 in the active site, and of Ala220 and Asn221 in the 220s loop, are labeled and shown as sticks, and alpha helix 7 and beta strand f are labeled. The dashed line indicates a distance short enough to permit bonding between the indicated functional groups. Coloring as in Fig 6.

A nearby region in 3D space, encompassing residues 181–191 (hereafter 180s loop) is resolved only in the Lys220Ala structure, and there only partially, with the backbone traced confidently through Arg182. Residues 183 to 191 can be traced with less confidence through weak electron density in both monomers of the Lys220Ala asymmetric unit. Residues 180 to 191 form an irregularly-structured segment lying directly above the active site (Fig 7). In the WT HsdR structure the backbone and sidechains of residues 182–190 are unresolved; in the Lys220Glu mutant residues 181–191 are unresolved; and in the Lys220Arg mutant residues 181–188 are unresolved. In these three structures, well-resolved residues flanking weak intervening density constrain the backbone of the unresolved segments of the 180s loop to a position similar to that observed in the Lys220Ala structure. This position lies directly above the nuclease active site. The 180s loop partially overlaps the QxxxY motif of HsdR, residues Gln179, Ile180, His181, Arg182 and Tyr183.

The resolvability of the 180s loop in the Lys220Ala structure appears to be related to the rotation of the helix-like segment bearing residues 220 and 221. This shift that brings Asn221 into the position of WT Lys220 places Ala220 on the opposite side of the helix-like segment, where its carbonyl group comes within hydrogen-bonding distance (3.2 Å) of the Arg182 guanidino group (Fig 7). Such an additional hydrogen bond might limit fluctuations of the 180s loop, and may explain why this region is resolved in the Lys220Ala mutant only. Although the helix-like segment is also rotated in the Lys220Glu structure, the region preceding that segment is poorly defined in all four molecules of the asymmetric unit, and the Glu220 sidechain is not resolved. High mobility in this region may limit contact with the 180s loop. The fact that resolution of the entire 220s loop responds to the identity of residue 220 suggests that the function of this region involves more of the loop than only residue 220 that makes contact with ATP. The results also suggest a previously unrecognized role for one of the non-conserved residues of the QxxxY motif, in this case Arg182.

## Discussion

The Type I restriction and modification systems are divided into four families based on gene hybridisation and antibody cross-reactivity [40, 41]. The families differ greatly in overall length of their HsdR subunits, making primary structure alignments across families unreliable and obscuring the equivalences of residues outside the three motifs bearing the catalytic residues of the endonuclease domain [42–44, 25].

No 3D structures of other representatives are available to date that can help to identify residue equivalences across families. Therefore only the five experimentally characterized members of the Type IC family [13], of which plasmid-encoded EcoR124 is the prototype, can be analysed with confidence. Within this group the overall sequence identity for the two members with the full length HsdR sequenced, EcoR124I and NgoAV, is 74% over all 1038 aligned residues. In the Type IC family a Lys residue is fully conserved in a position equivalent to Lys220 of EcoR124I HsdR. Although the number of characterized family members is small, the strict conservation of Lys220 is consistent with the role reported here.

The crystal structures reported here show that mutant Lys220Arg HsdR maintains contact to ATP similarly as does Lys220 of WT HsdR, whereas mutants Lys220Ala and Lys220Glu lose contact with ATP. These results suggest that positive charge at residue 220 is required to maintain contact with the ATP base edge. Hybrid quantum-mechanics/molecular mechanics calculations [32] show that the energy contribution of the Lys220-ATP contact is comparable to the contributions of the other contacts to the adenine ring, and is therefore not likely to be a fortuitous contact or an artifact of crystallization. The contribution of the Arg220-ATP contact is expected to be at least as great as that of Lys220, due to its similar electrostatics and the potential additional hydrogen bond to the ribose; comparable QM/MM calculations for the three mutants are ongoing but are beyond the scope of the present work.

The crystal structure of mutant Lys220Ala presented here reveals an alternative interaction of the 220s loop with the 180s loop, indicating that the two loops can interact and suggesting a role for Arg182 of the QxxxY motif in that interaction. Extrapolating this finding to WT HsdR suggests that conformations in which Lys220 contacts ATP may alternate with conformations in which the 220s loop contacts the 180s loop. This suggestion is strengthened by results from a series of molecular dynamics simulations of WT HsdR [32] that were initiated using the 180s loop conformation of the Lys220Ala mutant in which this loop is uniquely resolved, with *in silico* reversion of Ala220 to the WT Lys220 residue. When the Lys220-ATP contact is lost a local conformational change is observed in which the Lys220 side chain turns toward the adjacent Asp219 and forms a hydrogen bond with its side-chain carboxylate Oδ1 atom. This new contact is persistent once formed. The 220s loop moves in the direction of the 180s loop, bringing the backbone carbonyl Ο atom of Asn221 within hydrogen-bonding distance of the Arg182 guanidino Nε atom. A hydrogen bond is formed between these two atoms that persists for the entire simulation. This conformation of the 180s and 220s loops resembles the crystal structure of the Lys220Ala mutant, although in that case contact between the loops is made with the backbone of Ala220 rather than Asn221, reflecting the rotation of the helix-like segment in the mutant. In simulations in which Lys220-ATP contact is maintained the conformations of the 180s and 220s loops resemble the WT crystal structure. No contacts between the loops are observed, and the side chains of Arg182 and Lys220 come no closer than ~13.5 Å.

Thus, both the crystal structures and the MD results suggest that the 220s loop alternates between two dominant conformations, one in which it interacts with ATP and another in which it interacts with the 180s loop *via* Arg182. Because the 180s loop lies directly above the nuclease active site, the interaction between the two loops provides a path from ATP to the active site. This structural alternation of the loops could report on the ATP ligation status of the endonuclease/motor subunit, and may thus be involved in coupling of translocation and endonuclease activities. A possible model for the conformational signal transmission mechanism that is implied by the previous computational results, and is consistent with the present experimental data, is shown on Fig 2 of reference [32].

The MD simulations also reveal global conformational changes that are correlated with loss of the Lys220-ATP contact. Rotational movement of the entire endonuclease domain relative to the other three domains of HsdR is correlated with the conformation of the 180s and 220s loops. When the Lys220–ATP contact is engaged and the 180s and 220s loops are far apart, the endonuclease domain remains within the plane formed by the other three domains; the planar array observed in the crystal structures (Fig 1) represents the unrotated state of the domain. The limited movements of the endonuclease domain in this case are exemplified by the atomic distances between Asn221Cα and Arg182Cα, which are narrowly distributed (~10.8 ± ~0.4 A) even though the loops are too far apart to interact. On the other hand, in simulations in which the Lys220–ATP contact is lost, with close contact between the 180s and 220s loops and Lys220 interacting with Asp219, principal-components analysis of the MD results shows that the endonuclease domain rotates by ~18° out of the plane. With reference to Fig 1A the direction of rotation can be described as a motion around a screw axis close to the 220s loop with the lower right edge of the endonuclease domain moving counterclockwise up from the plane, with maximum displacement of ~10 Å corresponding to the mentioned out-of-the-plane angle of 18°.

These combined results from crystal structures and MD simulations suggest that the ATP ligation state of the enzyme might be communicated through the endonuclease/motor subunit by a cascade of linked local and global conformational changes. Such large-scale domain motions as observed in MD simulations are expected to be readily detected by the other enzyme subunits, and may represent a signal reporting the ATP ligation state of the endonuclease/motor subunit. The functional results of the present work indicate that DNA is not cleaved efficiently when the contact of residue 220 to ATP is lost permanently as in Lys220Ala or Lys220Glu, although all other functions of the enzyme remain WT-like. This finding suggests that the conformation of the 220s loop is likely to be the initiating signal in the mechanism by which stalled translocation triggers DNA cleavage. Thus it appears that alternating contacts of the 220s loop with ATP or with the 180s loop, and their correlated domain motions within the endonuclease/motor subunit, may be responsible for signaling the switch between translocating and endonucleolytic modes of the enzyme. These structural changes could also occur during each translocation step. Such an alternative mechanism could still encompass the current findings indicating that the endonuclease activity is dependent on ATP-dependent structural changes communicated by regions involving Lys220 and Arg182. In this view DNA cleavage could occur during the course of each translocation step with an extremely low probability, and the stalling of the translocation motor then would simply provide the kinetic opportunity for DNA cleavage, without specific activation of the endonuclease.

The 180s loop of EcoR124I HsdR contains the so-called QxxxY motif that is shared in some Type I enzymes and is also found in the recB helicases [44, 26]. This motif has not been assigned a clear function to date in either enzyme. Mutations Gln179Ala and Tyr183Ala in EcoR124I HsdR display long lag times for cleavage of the second DNA strand, suggesting an auxiliary role in DNA cleavage for the motif. MD simulations uncovered no interactions that could explain the functional effects of residues other than Arg182 [32]. In all characterized members of Type I family C enzymes an arginine residue is conserved in the QxxxY motif of HsdR (data not shown). In an alignment of 78 HsdR sequences that included also putative Type IC family members and more distantly related homologs of EcoR124I [26], the consensus sequence shows a polar side chain and hydrogen-bond donor in this position (Arg, Lys, or Thr). This consensus is specific for family IC; in family IB there is no consensus for the third x of the QxxxY motif corresponding to Arg182 of EcoR124I HsdR, and family IA does not have a clear QxxxY motif [26]. In MD simulations with a virtual Arg182Ala mutant [32] the 180s to 220s interloop distance is independent of the Lys220–ATP distance, and rotation of only ~ 11° is observed only in the first 40 ns before the system is equilibrated, indicating that the structural alternation observed in simulations with WT HsdR is decoupled. Functional tests demonstrate that the Arg182Ala mutant is restriction-deficient *in vivo* and defective for DNA cleavage *in vitro*, although ATPase activity is similar as in WT [32]. These results suggest that the Arg182 interaction with Asn221 upon loss of the Lys220–ATP contact may be a key role of the family IC QxxxY motif.

Like the Type I restriction enzymes, the recBCD enzymes switch between two major modes (3′→5′ nuclease and 5′→3′ nuclease) as a consequence of DNA recognition [45]. The finding that the QxxxY motif of EcoR124I HsdR may be involved in mode-switching by directing global motions *via* alternative contacts with a distant loop suggests the possibility that the recB QxxxY motif may have a previously undetected partner that facilitates its switch. Given the multisubunit nature of the recBCD complex, in contrast to EcoR124I HsdR where both translocation and endonuclease functions are housed on a single multidomain subunit, a putative partner of the recB QxxxY motif may be housed on a separate subunit, possibly complicating its detection and functional assignment; genetic approaches maybe useful to search for potential partners.

## Materials and Methods

### Bacterial strains, plasmids, microbiological techniques

*E*.*coli* strain JM109(DE3) [46] served for complementation analysis of restriction function *in vivo*. For positive and negative complementation assays the plasmids pKF650 carrying all three EcoR124II *hsd* genes and pACMS expressing only the EcoR124II methylase [47] were introduced into JM109(DE3) together with compatible plasmids carrying WT *HsdR* or each individual mutated *hsdR* gene. The virulent mutant of phage λ was used for testing of restriction and modification [48]. Phage buffer, complex LB medium and *in vivo* restriction and modification assays were as described [49, 33]. The solid medium is LB with agar added at 1.5%. Soft agar overlay is LB with agar added at 0.6%. Antibiotics were used at the following concentrations: ampicillin; 100 mg ml^-1^, chloramphenicol; 50 mg ml^-1^.

### Mutagenesis

Mutant primers (5’- CGCGAT**GC**AAACAGTTTTG -3’ for Lys220Ala (residue 220 codon underlined; mutated bases bold); 5’- CGCGATA**G**AAACAGTTTTG -3’ for Lys220Arg; 5’- CGCGAT**G**AAAACAGTTTTG -3’ for Lys220Glu) and reverse primer ~200 bp downstream (5’- GAACTCTTAATTTTCCACAG-3’) were used to generate mutant megaprimers in 50 μl PCR reactions containing 40 ng plasmid DNA pTrcR124 [11], 130 μM dNTPs, 10μM each primer, 1.5 u Pfu polymerase, and 1x Pfu polymerase buffer. PCR amplification conditions were 3 min 94°C, 15 x (0.5 min 94°C, 0.5 min 55°C, 0.5 min 72°C), 5 min 72°C. 1.5% agarose gel was used to visualize megaprimers. Megaprimers were cleaned up after gel extraction (QIAquick, Qiagen) and eluted in TE buffer, then used in 50 μl PCR reactions containing 60 ng template plasmid pTrcR124, 200 μM dNTPs, 1.25 μM megaprimer, 3 U Pfu polymerase, 40 μM MgSO4, 1x Pfu buffer. PCR amplification conditions were 3 min 94°C, 15 x (0.5 min 90°C, 9 min 72°C), 20 min 72°C. DpnI endonuclease cleavage (20 U), 37°C for 1h was used to degrade template DNA. *E*. *coli* TOP10 cells were transformed with the reaction mixtures. DNA sequencing using primers 5’-CTTACCGCTGGTACAAATC-3’ and 5’-CTGGTACGGTCGCATTAC-3’ confirmed the mutations.

### Protein expression and purification

EcoR124I methylase was purified from *E*. *coli* strain JM109(DE3) harboring plasmid pJS4M as described [50]. WT and mutated HsdR subunits produced from mutated plasmid pTrcR124 were purified as described [11]. The mutant HsdR subunits behaved as WT during purification (data not shown).

### Crystallization and data collection

Crystals of HsdR Lys220Arg were obtained by sitting-drop vapor diffusion as described for WT HsdR [51]. Single crystals were soaked in 25% (v/v) glycerol prior to diffraction and cooled to 100 K. Data were collected to 2.7 Å on beamline X13 at EMBL/DESY with a wavelength of 0.801 Å and a MAR 165 CCD detector with 0.5° oscillation. Data from 300 images were integrated and scaled with *MOSFLM* and *SCALA* from the CCP4 suite of programs [52].

Crystallization trials of HsdR Lys220Glu and Lys220Ala were carried out in CombiClover crystallization plates (Emerald Biosystems, Bainbridge Island, USA) using a sitting-drop vapor-diffusion at 20°C and 4°C, respectively. Crystals with similar dimensions of ~300×300×50 μm were obtained within one week

HsdR Lys220Glu was concentrated to 10 mg/ml in 20mM potassium phosphate buffer, pH 8.0, 50mM NaCl, 2mM ATP. Drops consisted of 6 μl protein, 2 μl reservoir solution and 0.8 μl 2M NH4F and were equilibrated against 400 μl reservoir solution (22% (w/v) PEG 4000, 0.1M MES buffer at pH 5.7). Crystals of ~300×300×50 μm were obtained within one week. For data collection, crystals were transferred with a nylon loop (Hampton Research) to a cryoprotectant consisting of the reservoir solution supplemented with 25% (w/v) glucose and immediately placed in a 100 K nitrogen-gas stream. Data collection to 2.99 Å resolution was performed on beamline X11 at EMBL/DESY with a wavelength of 0.815 Å and a MAR 555 (flat panel) detector with 0.5° oscillation. Data from 451 images were integrated and scaled with *XDS* and *XSCALE* [53].

HsdR Lys220Ala was concentrated to 6 mg/ml in 20mM Tris-HCl buffer, pH 7.6, 50mM NaCl, 2mM ATP. Propylamine was diluted with 0.2M monopotassium phosphate buffer pH 7.6 and added to the protein at final concentration ~ 6mM prior to crystallization. Protein solution (5 μl) was mixed with 2 μl reservoir solution (13% (w/v) PEG 4000, 22% glycerol, 0.08M MES buffer pH 5.9, 2mM DTT) and 0.7 μl 0.1M sodium bromide as an additive. Drops were equilibrated against 500 μl reservoir solution. Crystals were mounted in a nylon cryoloop, immersed in cryoprotectant (13% (w/v) PEG 4000, 25% glycerol in 0.08M MES buffer pH 5.9) for a few seconds and then flash-frozen in a stream of nitrogen gas at 100K. Data collection was performed to 2.84 Å resolution on beamline BW7B at EMBL/DESY on a MAR 345 detector with 0.5° oscillation angle. Data were integrated with *XDS*, an MTZ file was produced by *POINTLESS* and data were scaled in *SCALA*.

### Structure solution and refinement

Crystallographic data and statistics are compiled in Table 2. HsdR Lys220Arg crystals belong to space group P2_1_ with unit cell dimensions *a* = 87.05 Å, *b* = 124.35 Å, *c* = 128.01 Å, and *β* = 108.86°. The molecular mass of HsdR is 120 kDa, yielding a Matthews coefficient [54] of 2.72 Å^3^Da^-1^ (55% solvent content) with two molecules in the asymmetric unit. The structure was solved by molecular replacement using *MOLREP* [55] with wild type HsdR (PDB 2W00) [22] as the search model. First, a search was performed for the monomer. After the solution was found, the monomer was fixed and a search was conducted for the second molecule. The structure was subjected to rigid-body refinement. Solvent flattening, histogram matching, and NCS averaging were then applied to improve phases using the program *DM* [56]. Finally, restrained NCS and TLS refinement was performed in *REFMAC5* [57]. TLS groups were calculated by analyzing the spatial distribution of individual atomic thermal parameters using the TLSMD web server [58]. The final R_cryst_ and R_free_ are 25.17% and 29.23%, respectively.

HsdR Lys220Glu crystals also belong to space group P2_1_ but unit cell dimensions differ from HsdR Lys220Arg (*a* = 127.11 Å, *b* = 123.11 Å, *c* = 160.11 Å, and *β* = 111.48°). The crystals contain four molecules in the asymmetric unit with Matthew's coefficient 2.4 Å^3^Da^-1^ (49% solvent content). The structure was solved by molecular replacement in Auto-Rickshaw [59] using *MOLREP* with the structure of wild type HsdR (PDB 2W00) as the search model. Initial rigid-body, position, and B-factor refinement were performed in *CNS* [60]. Refinement was continued in *PHENIX* using NCS with secondary structure restraints applied. The final R_cryst_ and R_free_ are 25.55% and 29.65%, respectively.

HsdR Lys220Ala crystals belong to space group P2_1_ with unit cell dimensions similar to those of Lys220Arg crystals (*a* = 86.33 Å, *b* = 124.49 Å, *c* = 128.76 Å, and *β* = 108.31°). The crystals contain two molecules in the asymmetric unit with Matthew's coefficient of 2.73 Å^3^Da^-1^ and 55% solvent content. The structure was solved by molecular replacement using *MOLREP* implemented in Auto-Rickshaw [59] with WT HsdR (PDB 2W00) as the search model. Initial rigid-body, position, and B-factor refinement were performed in *CNS* [60]. Final refinement was carried out in *REFMAC* and included atomic coordinate and isotropic B-factor refinement with NCS restraints applied. The final R_cryst_ and R_free_ are 23.36% and 28.04%, respectively. Comprehensive validation of the models including MolProbity analysis was performed in *PHENIX* with 96% of residues found in Ramachandran favored regions. The outliers are associated with poorly defined electron densities.

### DNA cleavage assays

Cleavage assays on circular DNA with one recognition site [11] with reconstituted WT or mutant EcoR124I were conducted. Reactions contained 15 nM DNA (circular plasmid pRK described as pDRM-1R [50], restriction buffer (50 mM Tris-HCl, 50 mM NaCl, 10 mM MgCl_2_, 1 mM dithiothreitol, pH 8.0). HsdR was assembled with the cognate methylase to form the endonuclease complex before addition of DNA substrate. The enzyme was reconstituted from 15 nM WT methylase and 90 nM WT or mutant HsdR, which corresponds to DNA:enzyme ratio of 1:1, and methylase:HsdR ratio of 1:6. Reactions were initiated at 37°C by addition of ATP and S-adenosyl methionine to a final concentration of 4 mM and 0.2 mM, respectively. The chosen ratio of HsdR to methylase has been experimentally determined as the ratio with maximum enzyme activity, and the further increase of HsdR concentration does not show any further activity increase. Aliquots of 20 μl were removed at the indicated time points and the reaction was stopped by adding 0.25 vol of stop solution (3% SDS, 0.15M EDTA, 10% glycerol, 0.1% bromophenol blue) and heating to 65°C for 5 min. Cleavage products were resolved by agarose gel electrophoresis and quantified. The samples were analyzed on 1.2% (w/v) agarose gel at 5V/cm for 2h in Tris-acetate-EDTA buffer followed by visualization using ethidium bromide. Intensity of DNA bands was quantified by scanning the gels using ImageJ software (version 1.45s). Band densities were plotted and fitted to an exponential rise to maximum function (f = a*(1-exp(-λ*x))) using SigmaPlot Version 12.0 (Build 12.1.0.15). Fig 2 A shows a typical gel and Fig 2B includes the standard deviations from seven repetitions of the experiment for WT, Lys220Ala, Lys220Glu or six repetitions for Lys220Arg conducted with independently purified enzyme preparations.

### ATPase assays

ATPase activity was assayed in NEB2 buffer (10 mM Tris-HCl, 50 mM NaCl, 10 mM MgCl_2_, 1 mM dithiothreitol, pH 7.9) containing 15 nM methylase, 90 nM HsdR, and 60 nM of the circular plasmid used in the DNA cleavage assays. Reactions incubated at 37°C were initiated by addition of ATP to the indicated final concentrations. Aliquots were taken at the indicated time points and stopped by addition of SDS and EDTA. The concentration of inorganic phosphate (P_i_) released by ATP hydrolysis was measured by spectrophotometric quantification of a phosphomolybdate–malachite green complex [61] or by autoradiographic detection of radioactive phosphate. 40 μl of sample was mixed in ELISA microplates with 150 μl of malachite green reagent (5.72% (w/v) ammonium molybdate in 6 M HCl; 2.32% (w/v) polyvinyl alcohol; 0.0812% (w/v) aqueous malachite green; and water in a ratio of 1:1:2:2). After five minutes, plates were scanned at 620 nm in a microplate reader. The quantity of P_i_ was determined from a calibration curve derived from solutions of known P_i_ concentration using KH_2_PO_4_ as a standard.

For radioactivity-based ATPase assays, reactions were carried out in NEB2 buffer containing 10 mM Tris-HCl, pH 7.9, 10 mM MgCl_2_, 50 mM NaCl, 1 mM DTT, 15 nM methylase, 90 nM HsdR, and 90 nM plasmid DNA, containing one recognition site for EcoR124I (pRK). Reactions (40 μL) were started by addition of ATP mixture containing 0.16 μCi (0.0013 mM) [γ^32^P]-ATP to a final concentration of 2 mM and incubated at 37°C. Aliquots (4 μL) were taken at the indicated time points and stopped by adding 1% SDS. The hydrolyzed ^32^P_i_ was separated from [γ^32^P]-ATP by cellulose TLC in 0.4 M LiCl_2_, 1M formic acid [36] and the distribution of radioactivity between ^32^P_i_ and ATP was visualized using a Fujitsu 9000 scanner [37].

### DNA—binding assays

The 30-mer duplex was prepared by annealing equimolar concentration of complementary oligonucleotides (Fig 3A) and 5´end-labelled with [γ^32^P]-ATP using T4 polynucleotide kinase in kinase buffer and incubated for 30 min at 37°C. The unincorporated ATP was removed with QIAquick Nucleotide removal Kit (QIAGEN). DNA binding reactions were performed in a volume of 10 μl in a buffer consisting of 50 mM Tris (pH 8.0), 25 mM NaCl, 10 mM MgCl_2_, 1 mM DTT, and 10% (v/v) glycerol. 5 nM end-labelled DNA duplex was incubated with 40 nM WT methylase, and with *in vitro* assembled WT or mutant HsdR. Methylase/HsdR mixtures at a range of molar ratios were incubated at room temperature for 10 minutes and for a further 10 min after adding duplex DNA. Bound and unbound DNA were separated on 6% polyacrylamide non-denaturing TAE gels run at 4°C and 100V. Gels were dried under vacuum for 30 minutes at 80°C (Model 583 Gel dryer, Bio-Rad) and visualized using a Molecular Dynamics PhosphorImager (Model BAS 5000, Fuji). A repetition conducted with independently purified enzyme preparations gave identical results.

### DNA translocation assay

Translocation analysis was performed by a combination of two previously described triplex oligonucleotide displacement assays [38, 39] with some modifications. Plasmid pLKS5 carrying a triplex binding site 2093 bp downstream of the recognition sequence for enzyme EcoR124I and the triplex-forming-oligonucleotide TFO14 (5’ TTCTTTTCTTTCTTCTTTCTTT 3’) were used [39]. Covalently closed pLKS5 DNA and ^32^P-labelled TFO14 were mixed in equimolar amounts (50 nM) in MM buffer (25 mM MES, pH 5.5, 12.5 mM MgCl_2_) and incubated at 20°C overnight. The resulting triplex (10 nM) was pre-incubated with methylase (100 nM) and HsdR (250 nM) at 20°C in R buffer (50 mM Tris–HCl, pH 8.0, 10 mM MgCl_2_, 1 mM DTT) for 5 min. Reaction was initiated by addition of ATP to a final concentration of 4 mM. The reaction was quenched after 1-h incubation at 20°C by addition of GSMB buffer (15% (w/v) glucose, 3% (w/v) SDS, 250 mM MOPS, pH 5.5, 0.4 mg/ml bromophenol blue) and the reaction products were analysed by electrophoresis in 1.5% (w/v) agarose gels [40 mM Tris–acetate, pH 5.5, 5.0 mM sodium acetate and 1.0 mM MgCl_2_] at 10 V/cm for 3 h at 4°C. Wet gels were exposed on an imaging plate (BAS-IP MS 2025, Fuji film) and visualized in a Molecular Imager FX Pro Plus system (Bio Rad).

### Protein Data Bank accession numbers

Coordinates for EcoR124I HsdR mutants Lys220Arg, Lys220Glu, and Lys220Ala have been deposited in the Protein Data Bank with accession codes 4BE7, 4BEB, and 4BEC, respectively.
